# Follow-up after critical care

**DOI:** 10.1186/cc9953

**Published:** 2011-03-11

**Authors:** M Slattery, P Temblett, A Houghton, D Hope

**Affiliations:** 1 ABM University Trust, Swansea, UK

## Introduction

Many patients experience physical and psychological morbidity following a stay in critical care [1]. The National institute of Clinical Excellence (NICE UK) recommends access to follow-up and rehabilitation services for this patient group [2]. We aim to present 1 year's experience following the establishment of a follow-up service at our university teaching hospital.

## Methods

The multidisciplinary follow-up team consisted of a consultant in critical care, a senior nurse and a critical care physiotherapist. Patients completed a preclinic questionnaire followed by a semi-structured interview to identify potential issues. Twenty-four clinics took place over the 12-month period.

## Results

A total of 221 patients were recruited. Of the patients studied 26% attended the clinic and completed the evaluation questionnaire, 30% did not engage follow up services. We identified recurrent themes in both physical and nonphysical problems. Example physical problems include limited physical activities in 77%, with 54% of patients studied finding difficulties with activities of daily living. Alteration in taste, smell, hearing and vision modalities was frequently described. In terms of psychological morbidity, anxiety and post-traumatic stress symptoms seem to predominate. Significant numbers of patients retain memory of their ITU stay, with one-third in the form of flashback memories. Only 5% of patients studied returned to work.

## Conclusions

Our findings demonstrate that a wide variety of problems can be identified in an ICU follow-up clinic. The challenge now is to identify those groups of patients who will benefit most from follow-up, to develop effective rehabilitation programmes for these patients, and to find methods to increase patient participation.
